# A PRISMA-IPD systematic review and meta-analysis: does age and follow-up improve active range of motion of the wrist and forearm following pediatric upper extremity cerebral palsy surgery?

**DOI:** 10.3389/fsurg.2024.1150797

**Published:** 2024-02-20

**Authors:** Amy X. Song, Anthony Saad, Lauren Hutnik, Onrina Chandra, Aleksandra McGrath, Alice Chu

**Affiliations:** ^1^ Department of Orthopedic Surgery, Rutgers New Jersey Medical School, Newark, NJ, United States; ^2^ Department of Statistics, Rutgers University, New Brunswick, NJ, United States; ^3^ Department of Clinical Science, Faculty of Medicine, Umeå University, Umeå, Sweden; ^4^ Department of Surgical and Perioperative Sciences, Faculty of Medicine, Umeå University, Umeå, Sweden

**Keywords:** upper extremity, cerebral palsy, systematic review & meta-analysis, forearm (MeSH), wrist (MeSH), pediatric, PRISMA (Preferred reporting items for systematic reviews and meta-analysis), surgery

## Abstract

**Purpose:**

Surgical treatments such as tendon transfers and muscle lengthening play a significant role in cerebral palsy management,but timing of upper extremity cerebral palsy surgery remains controversial. This study systematically reviews the current literature and investigates the correlation between age at surgery and follow-up time with surgical outcomes in pediatric upper extremity cerebral palsy patients.

**Methods:**

A comprehensive search of PubMed, Cochrane, Web of Science, and CINAHL databases was performed from inception to July 2020 and articles were screened using PRISMA guidelines to include full-text, English papers. Data analysis was performed using itemized data points for age at surgery, follow-up length, and surgery outcomes, reported as changes in active forearm and wrist motion. A 3D linear model was performed, to analyze the relationship between age, follow-up length, and surgery outcomes.

**Results:**

A total of 3,855 papers were identified using the search terms and a total of 8 studies with itemized patient data (*n*=126) were included in the study. The studies overall possessed moderate bias according to the ROBINS-I scale. Regression analysis showed that age is a significant predictor of change (|t| > 2) in active forearm supination (Estimate = −2.3465, Std. Error = 1.0938, *t*-value= −2.145) and wrist flexion (Estimate = −2.8474, Std. Error = 1.0771, *t*-value = −2.643) post-intervention, with older individuals showing lesser improvements. The duration of follow-up is a significant predictor of improvement in forearm supination (Estimate = 0.3664, Std. Error = 0.1797, *t*-value = 2.039) and wrist extension (Estimate = 0.7747, Std. Error = 0.2750, *t*-value = 2.817). In contrast, forearm pronation (Estimate = −0.23756, Std. Error = 0.09648, *t*-value = −2.462) and wrist flexion (Estimate = −0.4243, Std. Error=0.1859, *t*-value = −2.282) have a significant negative association with follow-up time.

**Conclusion:**

These results suggest that there is significant correlation between the age and follow up after surgery with range of motion gains. Most notably, increased age at surgery had a significant negative correlation with select active range of motion postoperative outcomes. Future research should focus on identifying other factors that could affect results of surgical treatment in upper extremity.

## Introduction

Cerebral palsy is a group of postural and movement disorders, involving limitations in range of motion, limb deformities, and spasticity. Deformities of the upper extremity present as limited active forearm supination, internal shoulder rotation, forearm pronation contractures, thumb-in-palm deformities and finger deformities including finger flexion and swan neck deformities (1). The upper extremity is commonly involved in cerebral palsy, and surgery is reserved for less than 20% of pediatric patients, typically those with functionally impairing deformities, significant cosmetic concerns, or considerable pain (2). Mild cases can be treated with conservative options such as botulinum toxins, splinting, and therapy (3). Surgical interventions for the upper extremity include tendon lengthening, transfer, and joint stabilization. Studies have shown single-event, multi-level surgery to be effective in improving upper extremity positioning, active range of motion, and reducing spasticity (4, 5).

However, the timing of surgery is widely debated. Some authors claim that surgery should be deferred until patterns of upper extremity use can be evaluated and when patients can follow protocols, generally between 5 and 12 years old (6). Some researchers believe that patients may benefit from postponing surgery until after pubertal growth spurts to prevent recurrence of the defomity (7). Others argue that surgery should be performed early before irreversible fibrosis occurs, although fibrosis occurs at variable ages (8). Few studies investigate the relationship between age of upper limb surgery and functional outcomes and there is currently no universally accepted guideline dictating timing for optimal results.

Furthermore, postoperative follow-up time varies widely among studies. Previous studies have shown that long term follow-up may be associated with decreased patient satisfaction, as positive results may wane over time and result in less patient satisfaction with final results (9). Rutz et al. also found that the results of single-event multilevel surgery for correcting spastic diplegia were not durable and most patients required additional surgeries 4–6 years later (10). These findings may be attributed to the recurrence and new onset of deformities, given that children possess significant growth plasticity. For this reason, postoperative rehabilitation is necessary in preserving joint mobility and preventing relapse of deformities (11). Given the variability in postoperative outcomes, the clinical result following one year after surgery cannot be considered final and study outcomes may be limited by insufficient follow-up.

Characteristic upper extremity deformities include weakness in active forearm supination, wrist extension, or spastic wrist flexion (11, 12). Therefore, studies often report both active and passive range of motion of the forearm and wrist perioperatively to monitor for functional improvement of these joints. Range of movement measurements can be used to evaluate pre-existing muscle length, spasticity, and muscle tone to predict postoperative outcome (13). As a result, this study primarily studies active forearm and wrist range of motion as postoperative outcomes.

The authors sought to systematically review the current literature and investigate the correlation between age at surgery and follow-up time with surgical outcomes in pediatric upper extremity cerebral palsy patients. The determination of this association between age at surgery and outcome can allow for better timing and tailoring of surgery, for maximal improvements in upper extremity function and quality of life in pediatric cerebral palsy patients.

## Methods

### Search strategy

A comprehensive search of Pubmed (1946–2020), Cochrane Library (1946–2020), Cumulative Index to Nursing and Allied Health Literature (CINAHL) (1946–2020), Web of Science (1945–2020) was performed. Articles were screened using the guidelines of the Reporting Items for Systematic Reviews and Meta-Analysis for Individual Patient Data (PRISMA-IPD) to include full-text, papers in English (14). Exploded Medical Subject Heading (MeSH) terms and key words were as follows: cerebral palsy AND (upper extremity OR upper limb) AND (pediatric OR child) AND (treatment OR therapy OR intervention). Six reviewers searched through reference lists to identify studies missed by the electronic search and studies were removed.

### Study selection and selection criteria

Seven reviewers screened articles by titles and then abstracts to determine the relevance of each article. Each article was screened by two reviewers. The remaining articles were screened by accessing and reviewing full-texts for study eligibility. Any disagreements among the reviewers were discussed and resolved.

Published studies were included if they met the following criteria: (1) Study population consisted of at least 3 children or adolescents (0–21 years) diagnosed with cerebral palsy affecting the upper extremity. Studies with overlapping age groups were included if data of subjects aged 0–21 years was reported individually. (2) Study intervention was a form of surgery, including pronator teres rerouting, tendon transfers, and muscle releases. (3) The study reported surgical outcomes using active forearm or wrist range of motion. (4) The study reported individual data, follow-up time, and characteristics for each patient.

The following exclusion criteria was used: (1) The article was a review, systematic review, comment, letter, or protocol. (2) The study intervention involved synergistic treatments, such as combined surgical and nonsurgical treatment. (3) The study looked at outcomes other than active range of motion of the wrist and forearm. Cross-referencing was performed to identify papers that were missed in the original search.

### Data extraction

From each study, the following data points were extracted for the purpose of creating a preliminary database: surgical intervention, publication year, outcome measurements reported, study type, sample size of patients, mean age, follow-up length, and whether the data was displayed as individual data points or as an average. Once studies were determined to be relevant to the topic of the meta-analysis, screening was completed to include only those that reported active forearm and wrist range of motion as an outcome measurement.

For each eligible study to be included in the meta-analysis, individual data on the patient's age, sex, length of follow up, active range of motion in forearm supination, pronation and wrist extension and flexion were collected.

### Quality of evidence and bias assessment

Quality of evidence was assessed according to the American Academy for Cerebral Palsy and Developmental Medicine (AACPDM) Treatment Outcome Committee guidelines. Level of evidence was determined using the following factors: inclusion and exclusion criteria, adherence to intervention assignment, outcome measurements, blinding status, statistical calculation, dropout rate, and control for confounding variables (15).

Risk of bias was determined according to the Risk of Bias in Non-Randomized Studies of Interventions (ROBINS-I) assessment tool (16).

### Statistical methods

Data analysis via R Statistical Software was performed using itemized data points for age at surgery, follow-up length, and surgery outcomes. Surgical outcomes was measured by changes in active forearm supination and pronation and wrist extension and flexion, as this was most commonly reported as an objective means of determining perioperative joint laxity and extent of limb deformity. A 3D linear model was performed, to analyze the relationship between age, follow-up length, and surgery outcomes.

## Results

### Study selection

**Figure 1:**
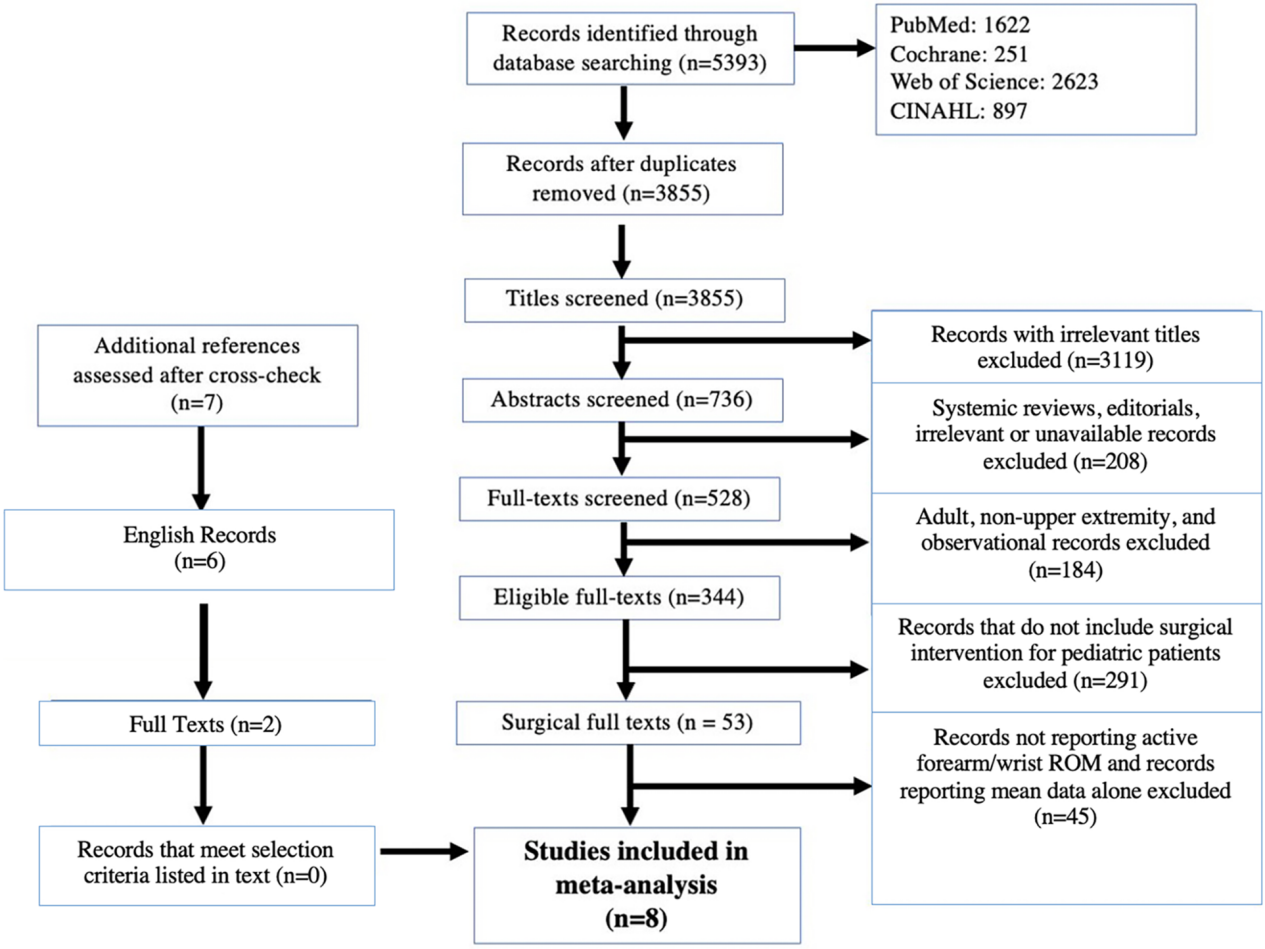
PRISMA—IPD flow diagram of included articles. PRISMA—IPD: Preferred reporting items for systematic review and meta-analyses of individual participant data.

Three thousand eight hundred fifty-five papers were identified using the search terms (Figure 1). Articles were screened by title and 736 abstracts were relevant. Next, texts were excluded if they were systemic reviews, editorials, irrelevant, or unavailable for access. Following abstract and title screening, there were 344 eligible full texts. No additional texts were identified through cross-referencing. Among the qualifying articles, 53 papers described upper extremity surgical interventions for pediatric patients. Of these, 13 reported active forearm and wrist range of motion. Another five papers were excluded for reporting mean data. Finally, eight studies with itemized patient data (*n* = 126) were included.

### Study characteristics, quality assessment, and bias

Seven of eight studies were case series, while one was a retrospective study (Table 1). Sample sizes ranged from four to 31 patients and follow-up time spanned 14–106 months. Surgical procedures included tendon transfer, pronator teres transfer, and muscle lengthening. Seven articles reported individual patient data including age at surgery, follow-up time, and changes in forearm pronation and supination, while 5 texts reported changes in wrist extension and flexion.

**Table 1:** Summary of included studies with type of study, sample size, mean age of surgery, mean follow-up time, type of surgical intervention, and study quality.

Study	Type of study	Country	Sample size	Mean age at surgery	Follow-up time(months)	Type of surgical intervention	Quality of study
Bunata et al., 2006	Case series	USA	31	8.4	39	Pronator teres rerouting	IV
Ho et al., 2014	Case series	Taiwan	17	12.6	46	Pronator teres rerouting	IV
Ponten et al., 2011	Case series	Sweden	18	11.11	7	Pronator teres rerouting, tendon transfer, muscle lengthening	IV
Ozkan et al., 2012	Retrospective Case series	Turkey	4	9.25	16	Tendon transfer	IV
Kreulen et al., 2004	Case series	Netherlands	10	16	14	Pronator teres rerouting, tendon transfer	V
Mifsud et al.,2020	Case series	India	13	14	14	Tendon transfer	IV
Helin et al., 2018	Case series	France	22	11.6	32.6	Pronator teres neurectomy	IV
Ponten et al., 2019	Case series	Sweden	12	10.75	106	Tendon transfer	VI

**Figure 2:**
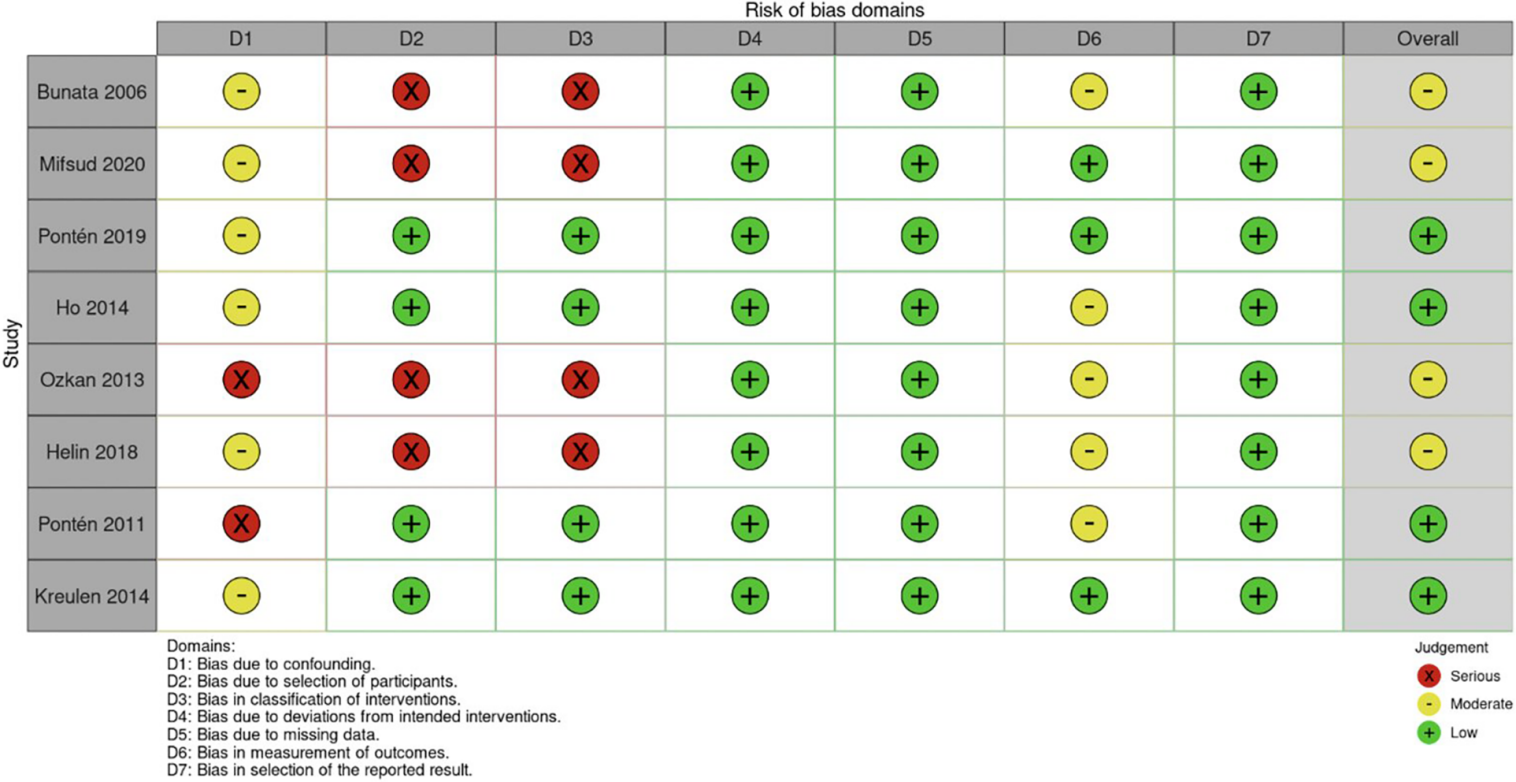
Risk of bias determined by I-ROBINS tool. I-ROBINS: Risk of bias in non-randomized studies of interventions.

According to AACPDM guidelines, six studies provided Level IV evidence, one study gave Level V evidence, and one study provided Level VI evidence (Table 1). As determined by the ROBINS-I scale, the studies overall showed moderate bias (Figure 2). The studies showed low bias in data selection and outcome reporting but demonstrated moderate or serious bias from confounding.

### Impact of age and follow-up time on functional outcome

Changes in active forearm supination (*n* = 125), pronation (*n* = 105), wrist extension (*n* = 66) and flexion (*n* = 50), were analyzed alongside surgical age and follow-up time. The mean age of surgery was 10.48 (SD 3.67) ± 4.62 years and average follow-up period was 23.26 (SD 19.99) months. The mean gain in forearm supination was 59.26° (SD 35.13) and average loss in forearm pronation was −15.22° (SD 18.64). Mean increase in wrist extension was 61.76° (SD 38.28) and average loss of wrist flexion was −24.18° (SD 25.50).

We ran a regression analysis the changes in each forearm and wrist movement on patient age and the duration of follow-up, categorizing age into groups to account for non-linear age effects. The analysis was based on data grouped into 5 age categories between ages 0 and 21 years. The 3D linear mixed model, fitted using REML (Restricted Maximum Likelihood), evaluated the relationship between each individual forearm and wrist and two key predictors: age (in years) and follow-up duration (in months), with random intercepts for different age categories.

**Figure 3:**
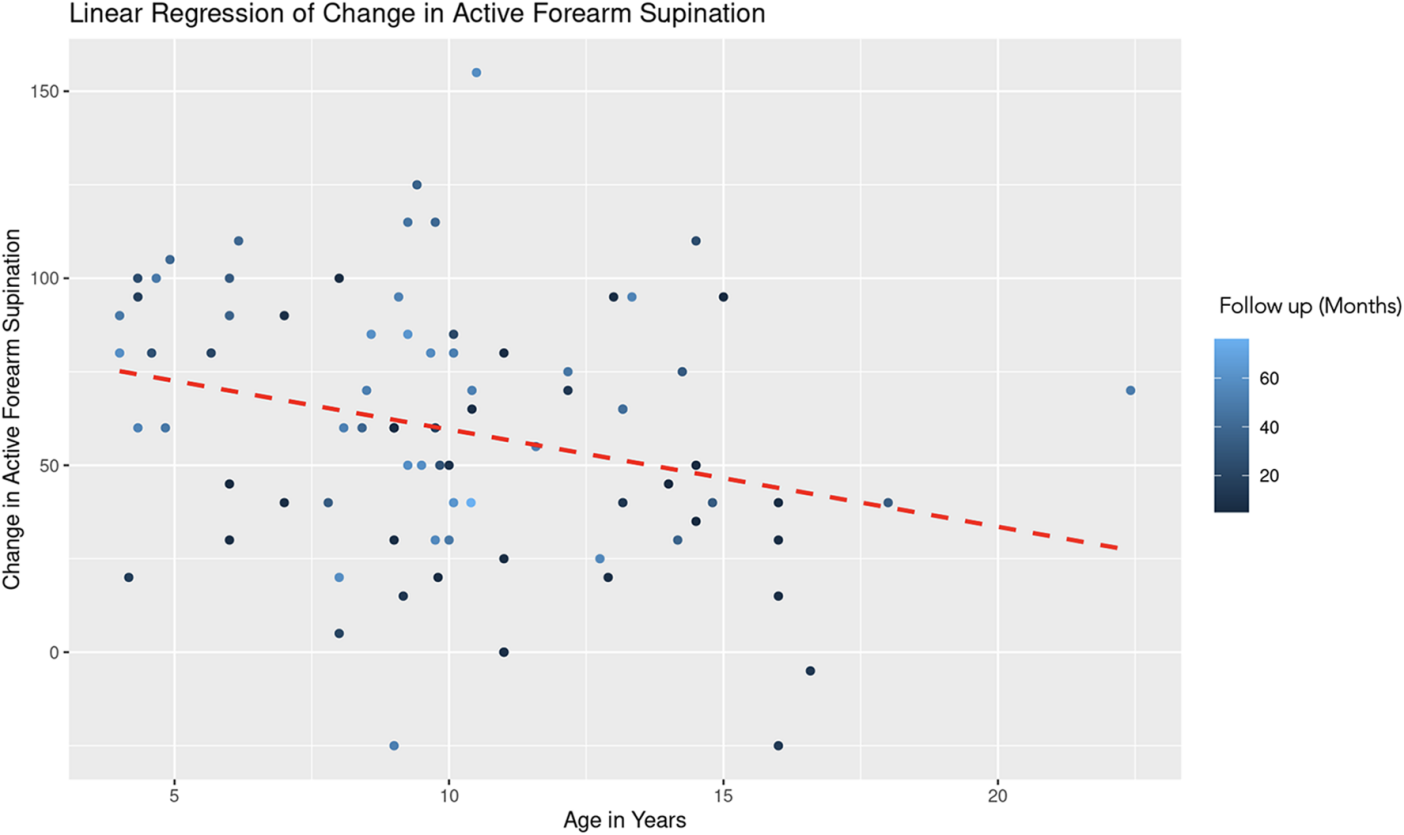
Linear regression of change in active forearm supination vs. age. A dashed red line indicates the linear regression fit, which shows a negative trend. With each additional year in age, the change in active forearm supination decreases by 2.35 degrees, controlling for follow-up time. The color gradient represents follow up time in months.

**Figure 4:**
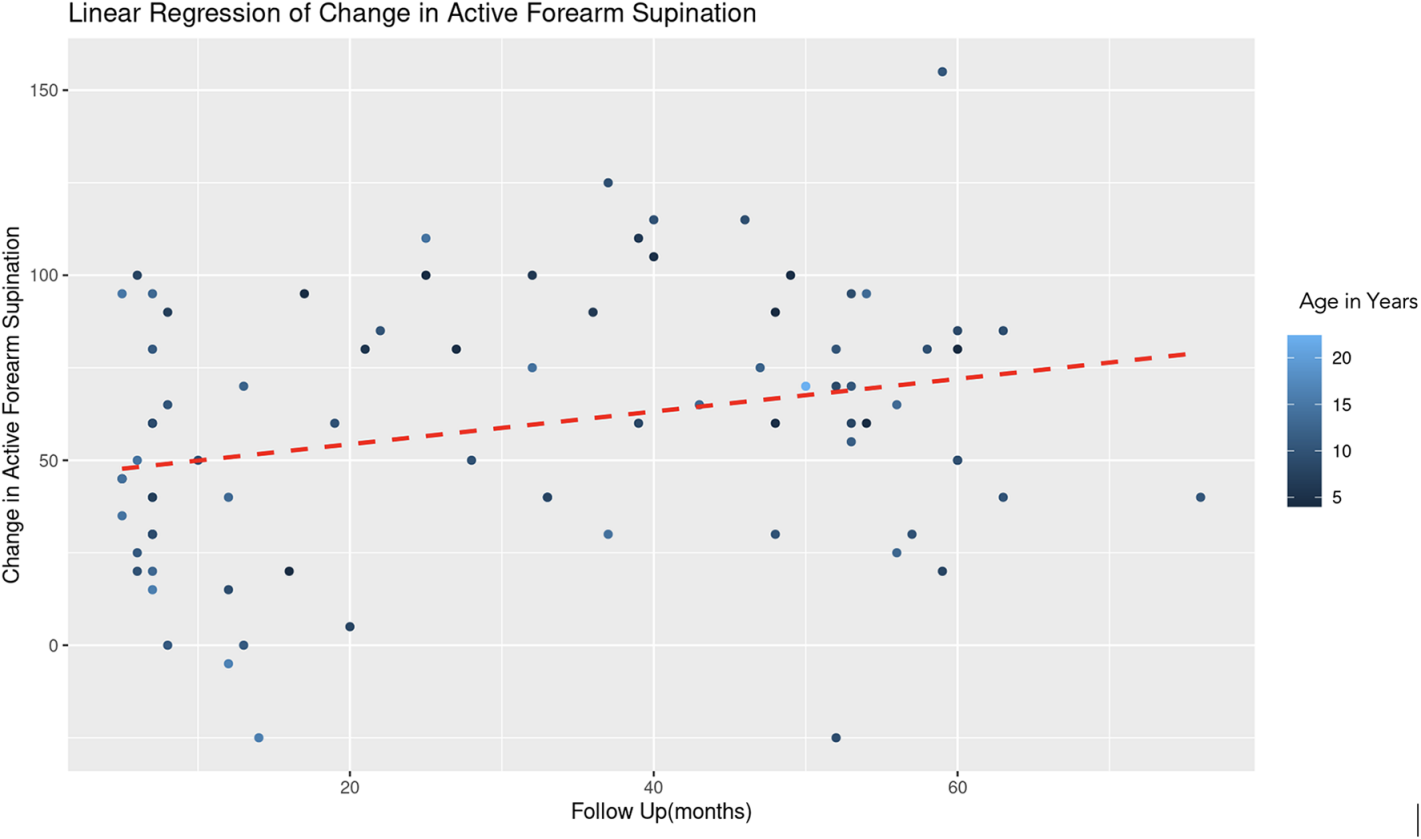
Linear regression of change in active forearm supination vs. follow up time. A dashed red line indicates the linear regression fit, which shows a positive trend. With each additional month of follow-up, there is an average increase of 0.37 degrees in the change of active forearm supination, controlling for age. The color gradient represents age in years.

For the regression model which evaluated active forearm supination, the estimated coefficient for age was −2.35 with a standard error of 1.09, yielding a t-value of −2.15. After controlling for follow up time, this result is statistically significant (|t| > 2) and indicates that for each additional year in age, the change in active forearm supination decreases by 2.35° (Figure 3). This suggests a negative association between age and the degree of improvement in forearm supination. The coefficient for follow-up duration was 0.37 with a standard error of 0.18, and a *t*-value of 2.04. This indicates a statistically significant positive relationship, suggesting that with each additional month of follow-up, there is an average increase of 0.37° in the change of active forearm supination, after adjusting for age (Figure 4). A REML criterion of 789.90 with well-distributed residuals indicates a robust model fit.

**Figure 5:**
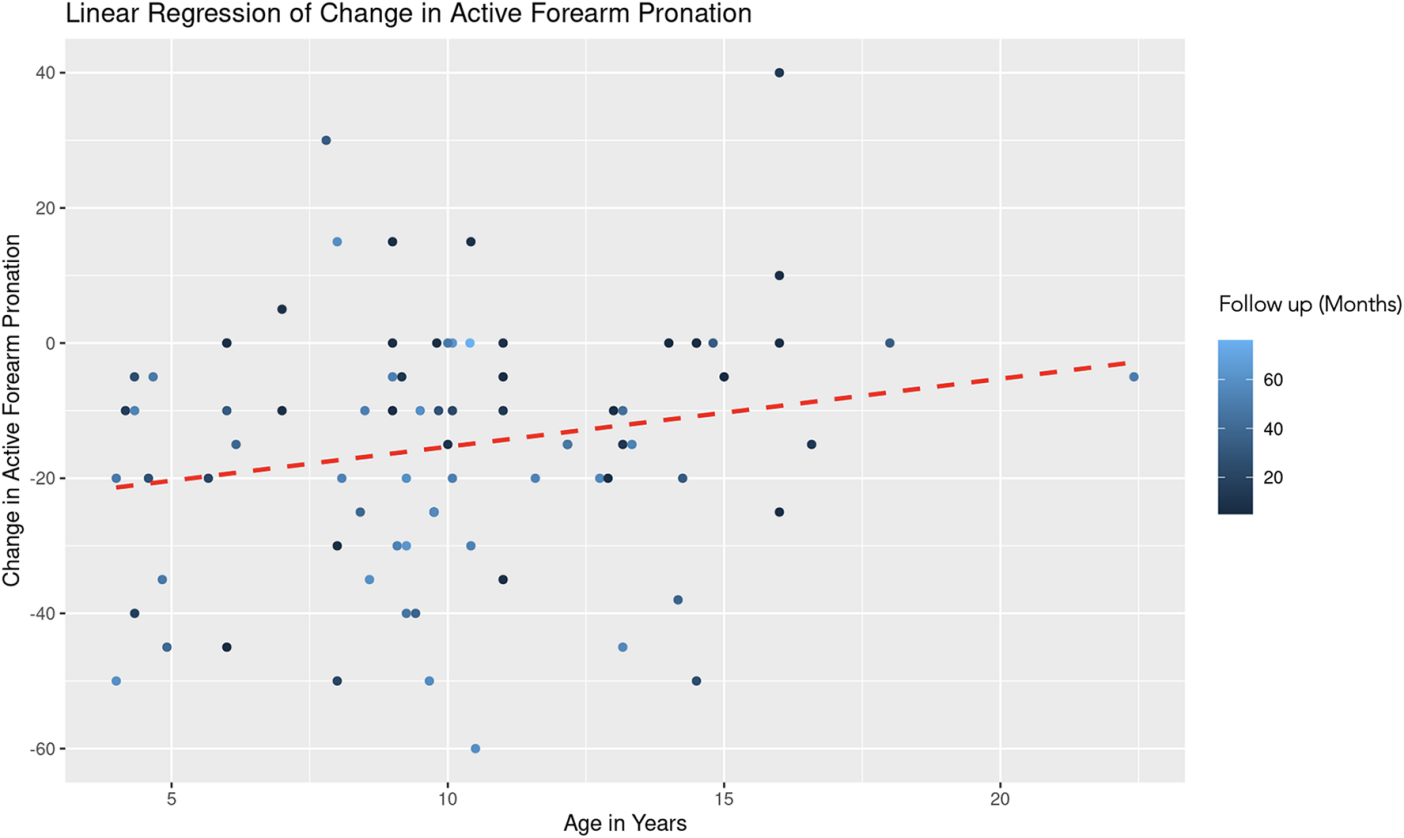
Linear regression of change in active forearm pronation vs. age. A dashed red line indicates the linear regression fit, which shows a positive trend. With each additional year in age, the change in active forearm supination increases by 0.78 degrees, controlling for follow-up time. The color gradient represents follow up time in months.

**Figure 6:**
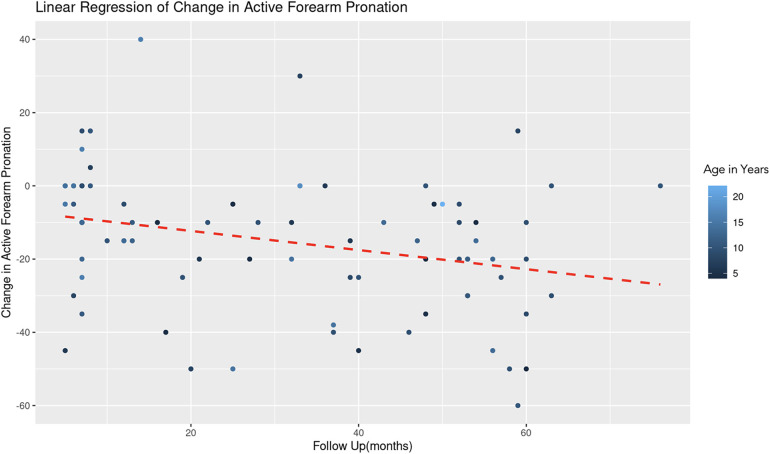
Linear regression of change in active forearm pronation vs. follow up time. A dashed red line indicates the linear regression fit, which shows a negative trend. With each additional month of follow-up, there is an average decrease of 0.24 degrees in the change of active forearm supination, controlling for age. The color gradient represents age in years.

A similar REML analysis regressing change in pronation on patient age and the duration of follow-up, reveals that there was no significant variability in forearm pronation change across different age groups. This indicates that the categorization of age might not contribute additional explanatory power to the model. The fixed effect of age (years) is estimated at 0.78 with a standard error of 0.54, resulting in a t-value of 1.44 (Figure 5). This suggests a positive association between age and change in forearm pronation, although this effect was not statistically significant (|t| < 2). The effect of follow-up time (months) showed a negative association with the change in forearm pronation (Estimate = −0.24, Std. Error = 0.10, *t*-value = −2.46). This indicates that longer follow-up durations are associated with a decrease in forearm pronation, and this effect was statistically significant (Figure 6).

**Figure 7:**
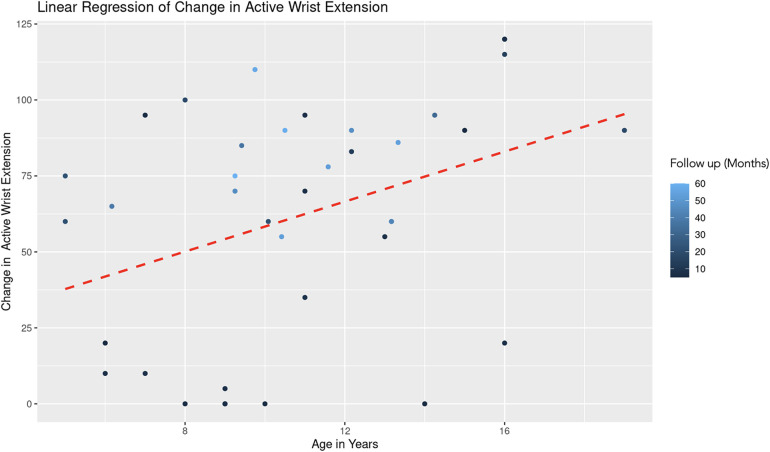
Linear regression of change in active wrist extension vs. age. A dashed red line indicates the linear regression fit, which shows a positive trend. With each additional year in age, the change in active forearm supination increases by 3.10 degrees, controlling for follow-up time. The color gradient represents follow up time in months.

**Figure 8:**
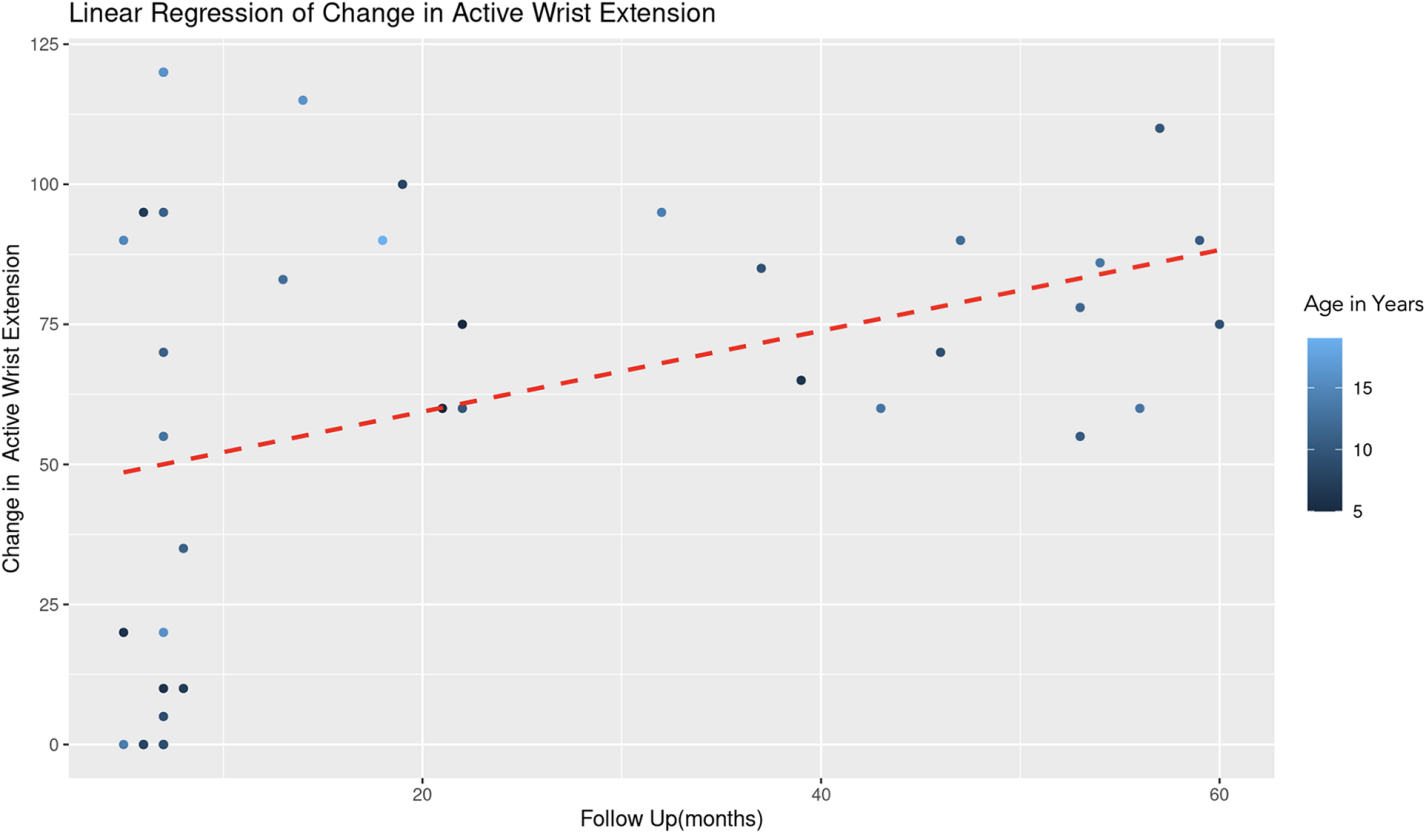
Linear regression of change in active wrist extension vs. follow up time. With each additional month of follow-up, there is an average increase of 0.77 degrees in the change of active forearm supination, controlling for age. The color gradient represents age in years.

The REML analysis regressing change in active wrist extension on patient age and the duration of follow-up revealed that the random effects associated with different age categories showed a significant variance (Variance = 178.70, Std. Dev. = 13.37). This indicates notable differences in the baseline level of wrist extension change across age groups. Age demonstrated a positive association with wrist extension change (Estimate = 3.10, Std. Error = 2.06, *t*-value = 1.51). However, this association was not statistically significant (Figure 7). Follow-up duration showed a significant positive effect (Estimate = 0.77, Std. Error = 0.28, *t*-value = 2.82). This suggests a statistically significant association, with longer follow-up durations correlating with greater improvements in wrist extension (Figure 8). The model converged successfully with a REML criterion at 363.90, indicating an adequate fit. The scaled residuals indicated no major violations of the model assumptions.

**Figure 9:**
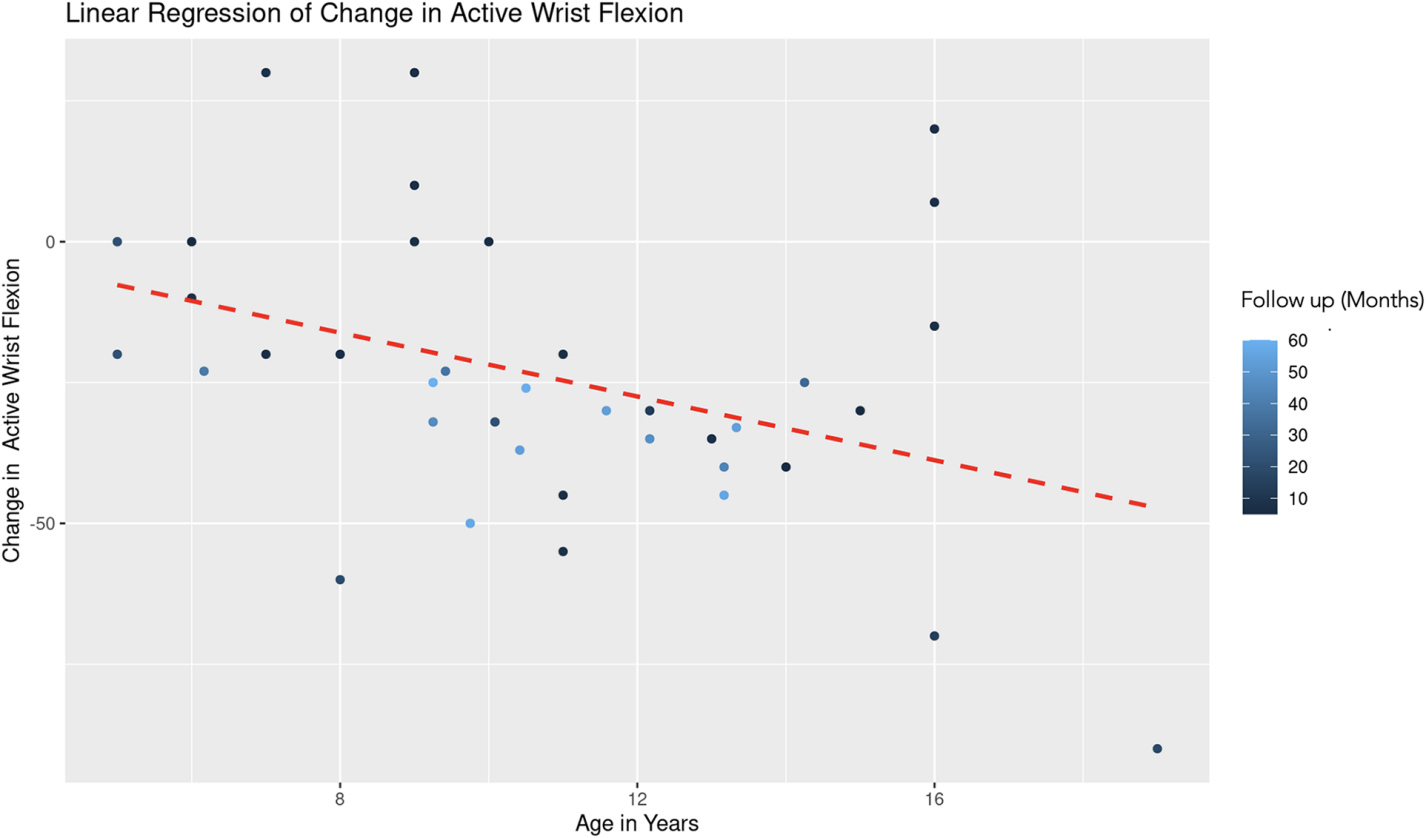
Linear regression of change in active wrist flexion vs. age. A dashed red line indicates the linear regression fit, which shows a negative trend. With each additional year in age, the change in active forearm supination decreases by 2.85 degrees, controlling for follow-up time. The color gradient represents follow up time in months.

**Figure 10:**
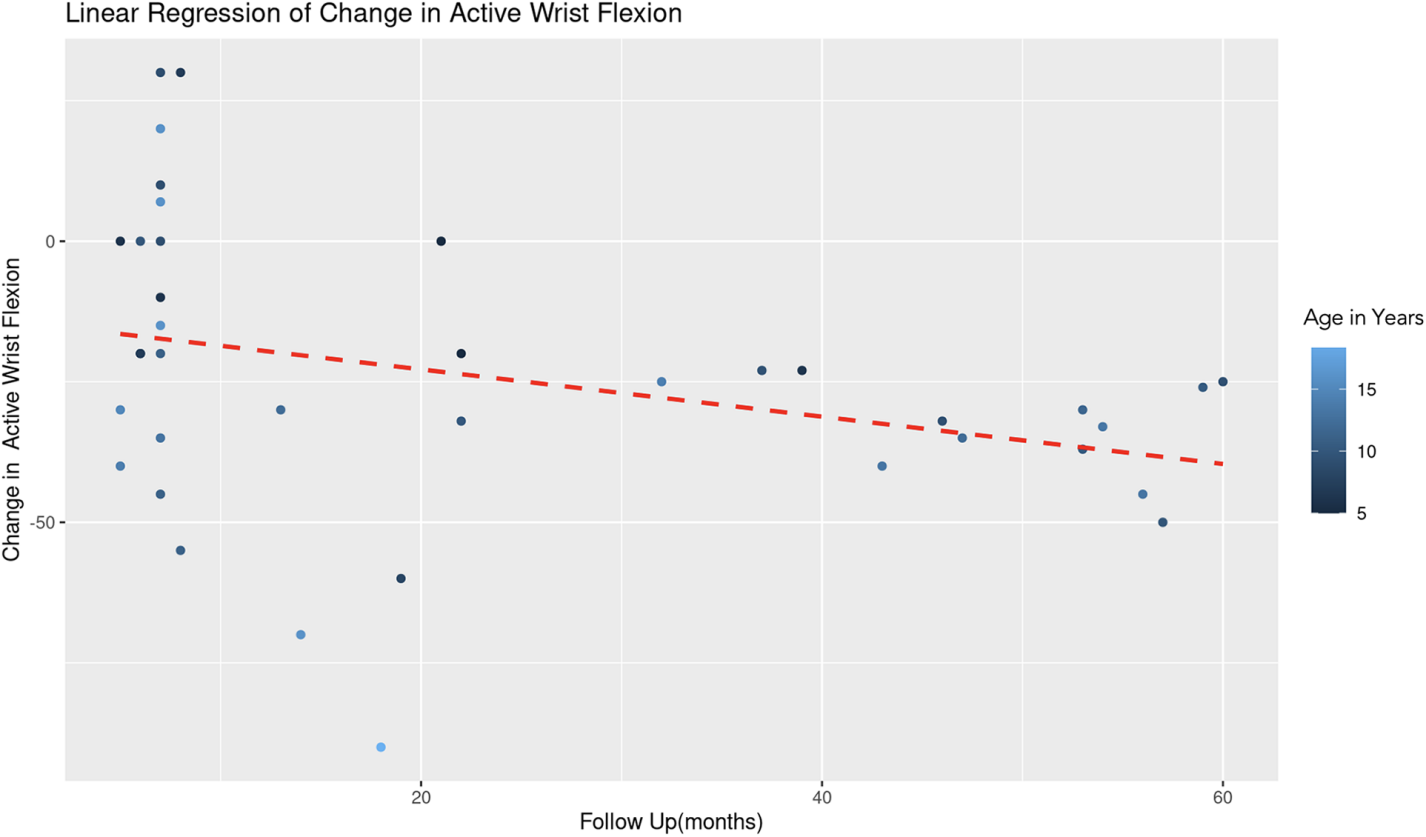
Linear regression of change in active wrist flexion vs. follow up time. A dashed red line indicates the linear regression fit, which shows a negative trend. With each additional month of follow-up, there is an average decrease of 0.42 degrees in the change of active forearm supination, controlling for age. The color gradient represents age in years.

The REML analysis regressing change in active wrist flexion on patient age and the duration of follow-up revealed that age demonstrated a significant negative effect on wrist flexion change (Estimate = −2.85, Std. Error = 1.08, *t*-value = −2.64). This indicates that an increase in age is associated with a decrease in the improvement of wrist flexion (Figure 9). Follow-up duration also showed a significant negative impact (Estimate = −0.42, Std. Error = 0.19, *t*-value = −2.28), suggesting that longer follow-up periods are associated with lesser improvement in wrist flexion (Figure 10). The scaled residuals indicated a reasonable fit of the model. However, since there was no significant variability in the change in wrist flexion across different age groups, the categorization of age may not be necessary for the model.

Overall, our findings indicate that age is a significant predictor of change in active forearm supination and wrist flexion post-intervention, with older individuals showing lesser improvement. There was no significant association between forearm pronation and wrist extension with age. Moreover, the duration of follow-up is a significant predictor of improvement in forearm supination and wrist extension, with longer follow-up periods being associated with greater changes in these measurements. In contrast, forearm pronation and wrist flexion have a significant negative association with follow-up time, with longer follow-up periods being associated with smaller changes in these measurements. No associations were identified between patient sex and outcomes.

## Discussion

The age of surgical intervention remains controversial, given that few studies exist examining the effect of age at surgery on pediatric functional outcomes and current literature is conflicting. For instance, a study of 61 patients between the ages of 5 and 41 found that there were no significant differences in outcomes among the different age groups, as each age group appeared to benefit from improvements in range of motion following surgery (17). Mortenson et al. reported that younger patients had greater functional improvements in upper extremity function following selective dorsal rhizotomy (18). However, other studies have found that older children aged 10–12 experienced the greatest improvement in gait following surgical treatment (19, 20). As a result, there currently exist no definitive guidelines regarding optimal timing of surgery.

This systematic review found that age of surgery may affect outcomes of surgical interventions for pediatric cerebral palsy, as measured by active forearm and wrist range of motion. Increased age at surgery had a significant correlation with both decreased forearm supination and decreased wrist flexion. These findings suggest that earlier surgical intervention might yield better outcomes. In the lower extremity, previous studies have found that older children showed less postoperative gain and younger age of surgery served as a predictor of improvement in gross motor function (8, 21). This may be due to the effect of contracture development in late childhood, during which puberty and growth spurts occur (22, 23). As a result, older children with contractures may be less amenable to surgery to begin with and would have limited gains in range of motion following surgery. For instance, Patterson et al. suggests that children under 13 years old receiving tendon transfer surgeries may be at increased risk of developing postoperative deformities with the imminent onset of puberty (24). Furthermore, younger patients may have better compliance with postoperative rehabilitation, while older children may be more preoccupied with school activities. As postoperative range of motion exercises are crucial in preventing recurrent deformity and maintaining correction, lack of follow-up among older children can be detrimental for surgery outcomes (11). Overall, this systematic review seems to align with the conclusions suggested by these studies.

There are other studies which are proponents of delaying surgery, for example showing that deferring surgery of the lower extremity until older age yielded better long-term results (20). One advantage of delaying surgery may be that early surgical correction can lead to recurrence of the deformity and calcaneus gait (25). Furthermore, some argue that there is a higher risk of over or under-correction in younger patients with greater growth plasticity than older children (26). However, the recurrence of upper extremity deformities is not well documented in literature and more studies are needed to elucidate such risks of early surgical intervention.

This systematic review also found that there was a significant positive association between follow-up length of the study with improvements in active wrist extension and forearm supination. The gains in upper extremity function associated with long-term follow up may be explained by maintenance of intensive rehabilitation and increased use of the affected limb. In contrast, this review found a significant negative association between follow-up length of the study with improvements in active wrist flexion and forearm pronation. This is in line with current literature which suggests that gains in upper extremity function are most prominent in short-term follow up after surgery, with plateau of improvement or return to pre-operative function in the long-term. For instance, Pontén et al. found that arm function improved significantly in the first 7 months of follow-up but had returned to the same level as prior to surgery by 9 years of follow-up (27). Similarly, Nylander et al. reported that following surgery, improvement in upper extremity dysfunction was most prominent in the initial 6 months of follow-up, with no further improvements at long-term follow-up (28). However, these study findings may be due to children undergoing the most vigorous of their rehabilitation regimens immediately following surgery, so that postoperative outcomes are maximal in the short-term.

The findings reported in this systematic review should be interpreted in the context of its limitations. The reported outcome measurement was the active range of motion of the forearm and wrist, which is subject to measurement error. Furthermore, while range of motion is an objective outcome, it only describes the mobility of a joint. Although having adequate and free movement of a joint is necessary for the completion of everyday activities, this outcome does not directly characterize other components of upper limb function such as spasticity, dexterity, strength, or patient satisfaction. As a result, improvements in joint range of motion may not translate to improved postoperative functionality or quality of life at home. Further studies are needed to investigate patient reported outcome measures such as the Pediatric Outcomes Data Collection Instrument (PODCI), which were not reported among our included studies. Furthermore, some studies did not individually list the surgeries performed. As some procedures such as the flexor carpi ulnaris to the extensor carpi radialis brevis tendon transfer may particularly improve forearm supination, future studies are needed to individually assess the impact of varying surgical techniques.

In conclusion, this systematic review examined the active range of motion outcomes of forearm and wrist surgery in children with cerebral palsy and found significant correlations between age and length of follow up, with postoperative outcomes. Most notably, this review found that increased age at surgery had a significant negative correlation with select active range of motion postoperative outcomes. As this study was limited to investigating reported data, future research should focus on identifying other factors that could affect results of surgical treatment in upper extremity. Designing and publishing future studies so that individual patient data is available to scientists aiming to review it systematically would be a step towards uncovering how to best utilize surgery for this group of patients.

## Data Availability

The original contributions presented in the study are included in the article/supplementary materials, further inquiries can be directed to AS, as2851@njms.rutgers.edu.
